# Unprecedented high insulin secretion in a healthy human subject after intravenous glucagon-like peptide-1: a case report

**DOI:** 10.1186/1756-0500-7-326

**Published:** 2014-05-31

**Authors:** Filip K Knop, Asger Lund, Sten Madsbad, Jens J Holst, Thure Krarup, Tina Vilsbøll

**Affiliations:** 1 Diabetes Research Division, Department of Medicine, Gentofte Hospital, University of Copenhagen, Copenhagen, Denmark; 2 Department of Endocrinology, Hvidovre Hospital, University of Copenhagen, Hvidovre, Denmark; 3 Department of Biomedical Sciences, Faculty of Health Sciences, University of Copenhagen, Copenhagen, Denmark; 4 Department of Endocrinology, Bispebjerg Hospital, University of Copenhagen, Copenhagen, Denmark

**Keywords:** Incretin hormones, Glucagon-like peptide-1 (GLP-1), Glucose-dependent insulinotropic polypeptide (GIP), Insulin

## Abstract

**Background:**

The gut-derived incretin hormones, glucose-dependent insulinotropic polypeptide and glucagon-like peptide-1, are released in response to ingestion of nutrients. Both hormones are highly insulinotropic in strictly glucose-dependent fashions and glucagon-like peptide-1 is often referred to as one of the most insulinotropic substances known.

**Case presentation:**

Plasma insulin and C-peptide concentrations were measured in a healthy Caucasian male (age: 53 years; body mass index: 28.6 kg/m^2^; fasting plasma glucose: 5.7 mM; 2 h plasma glucose value following 75 g-oral glucose tolerance test: 3.5 mM; glycated haemoglobin A_1c_: 5.5%) during glucagon (1 mg) and meal (2,370 kJ) tests, and during two 2 h 15 mM-hyperglycaemic clamps with continuous intravenous infusion of glucagon-like peptide-1 (1 pmol/kg/min) and glucose-dependent insulinotropic polypeptide (4 pmol/kg/min), respectively. Normal insulin and C-peptide responses were observed during meal test (peak concentrations: 300 and 3,278 pM) and glucagon test (peak concentrations: 250 and 2,483 pM). During the hyperglycaemic clamp with continuous intravenous infusion of GLP-1 the subject exhibited plasma insulin and C-peptide concentrations of 13,770 and 22,380 pM, respectively.

**Conclusions:**

To our knowledge insulin and C-peptide concentrations of these magnitudes have never been reported. Thus, the present data support the view that glucagon-like peptide-1 is one of the most insulinotropic substances known.

## Background

The two incretin hormones, glucose-dependent insulinotropic polypeptide (GIP) and glucagon-like peptide-1 (GLP-1), are secreted from endocrine mucosal cells in the gastrointestinal tract (K and L cells, respectively) in response to ingestion of nutrients. Both hormones are insulinotropic in strictly glucose-dependent fashions [1].

A priori, the insulinotropic actions of GIP and GLP-1 would be expected to be similar: The receptors of the two incretin hormones are closely related and belong to the same branch of the superfamily of type 2 G protein-coupled receptors [2], and, furthermore, both receptors couple to adenylate cyclase and their insulinotropic actions depend to a large extent on the intracellular accumulation of cyclic AMP (cAMP). Additionally, exogenously delivered GIP and GLP-1 have been found to be equally insulinotropic at physiological plasma concentrations and to contribute equally to the incretin effect in healthy subjects [3]. However, physiological plasma concentrations of GIP are 2 to 5 fold higher compared to GLP-1 concentrations (during fasting conditions and in the postprandial state). Therefore, GLP-1 has often been cited as one of the most insulinotropic substances known [1,4-7].

Here we present a case of unprecedented high insulin and C-peptide responses in a healthy subject following intravenous (*iv*) administration of GLP-1 and discuss the existing literature on maximal beta cell secretory response tests and on mechanisms underlying the insulinotropic effects of the incretin hormones.

## Case presentation

### Subject

A healthy male Caucasian (age: 53 years; body weight: 90.7 kg; body mass index: 28.6 kg/m^2^) with normal glucose homeostasis (fasting plasma glucose: 5.9 mM; 2 h plasma glucose value following 75 g-oral glucose tolerance test: 3.5 mM; glycated haemoglobin A_1c_: 5.5%) and without any family history of diabetes was examined. Insulin resistance according to the homeostasis model assessment [8] was 1.65. He had no significant past medical history, took no medication and clinical examination was normal. All standard investigations including blood pressure and standard biochemical parameters in urine and blood were normal. He agreed to participate (verbal and written consent) after receiving oral and written information. The examinations were approved by the Scientific-Ethical Committee of the County of Copenhagen (journal no.: KA 97196 m) and conducted according to the principles of the Helsinki Declaration II.

### Methods

The subject participated in a study evaluating the insulinotropic power of supra-physiological doses of GLP-1 as compared to glucagon, GIP, and physiological conditions in healthy subjects [9]. He was examined on 4 different days (day 1 through 4) separated by minimum 48 hours. On each occasion the subject was studied after a 10 h-fast, including liquids, with a cannula inserted in a dorsal hand vein for collection of blood samples. On day 2, 3 and 4, another cannula was inserted into the contra-lateral cubital vein for infusion of glucagon (day 2) and glucose plus incretin hormones (day 3 and 4).

#### Meal test (day 1)

Blood was drawn regularly after a standard breakfast meal ingested during 15 min. The meal comprised 2,370 kJ (566 kcal) and was composed of 34% fat, 47% carbohydrate, and 19% protein.

#### Glucagon test (day 2)

Blood was sampled 15, 10 and 0 min before and 2, 3, 4, 6, 8, 10, 15, 20, 30, 45 min after *iv* bolus of 1 mg (=287 nmol) biosynthetic glucagon (GlucaGen, Novo Nordisk, Bagsværd, Denmark) in 1 ml of sterile water.

#### Hyperglycaemic clamps + GLP-1 (day 3) or GIP (day 4)

At time 0 min, a bolus of 50% glucose (w/v) was infused during 1 min to increase plasma glucose to 15 mM. Plasma glucose was kept at 15 mM by continuous infusion of glucose, which was adjusted every 5 min according to bedside measurements of plasma glucose. After 3 min, a continuous infusion of 1 pmol GLP-1/kg/min (day 3) or 4 pmol GIP/kg/min (day 4) was initiated. Blood was sampled 15, 10 and 0 min before and 5, 10, 15, 20, 25, 30, 45, 60, 75, 90, 105 and 120 min after elevation of plasma glucose.

An additional experimental day consisting of a 2 h 15 mM-hyperglycaemic clamp with continuous infusion of saline was designed in order to evaluate the insulin response to hyperglycaemia without incretin hormone infusion. However, the subject withdrew his consent due to the symptoms of hypoglycaemia experienced following termination of the hyperglycaemic clamp with infusion of GLP-1 (see below). Thus, we found it unethical to utilize the present design in further evaluations of beta cell function in healthy subjects [9].

On all experimental days, blood was sampled into tubes containing heparin or EDTA (6 mM) plus aprotinin (500 KIU/ml blood; Trasylol, Bayer, Leverkusen, Germany) and a specific dipeptidyl peptidase 4 (DPP-4) inhibitor (valine-pyrrolidide; 0.01 mM, final concentration; a gift from Novo Nordisk A/S Bagsværd, Denmark) for hormone analyses. These tubes were immediately cooled on ice and centrifuged for 20 min at 1,200 *g* and 4°C. Plasma was stored at -20°C until analysis. For bedside measurements of plasma glucose, blood was distributed into fluoride tubes and centrifuged immediately for 2 min at 7,400 *g* and room temperature.

### Analyses

Plasma glucose concentrations were measured during the experiments by the glucose oxidase method (Yellow Springs Instrument Model YSI 2300 STAT plus analyzer; YSI Inc., Yellow Springs, Ohio, USA).

Plasma samples were assayed for total GLP-1 immunoreactivity using antiserum no. 89390, which is specific for the C-terminal of the GLP-1 molecule and reacts equally with intact GLP-1 and the primary (N-terminally truncated) metabolite.

Total GIP was measured using the C-terminally directed antiserum R65, which reacts fully with intact GIP and the N-terminally truncated metabolite.

Plasma insulin concentrations were measured using commercial ELISA kits (Dako, Copenhagen, Denmark).

Plasma C-peptide concentrations were determined as described by Heding *et al. *[10] employing the polyclonal antibody M1230.

### Calculations and statistics

Area under the curve (AUC) values were calculated using the trapezoidal rule and data is presented using standard descriptive statistics.

## Results

### Glucose

**Figure 1:**
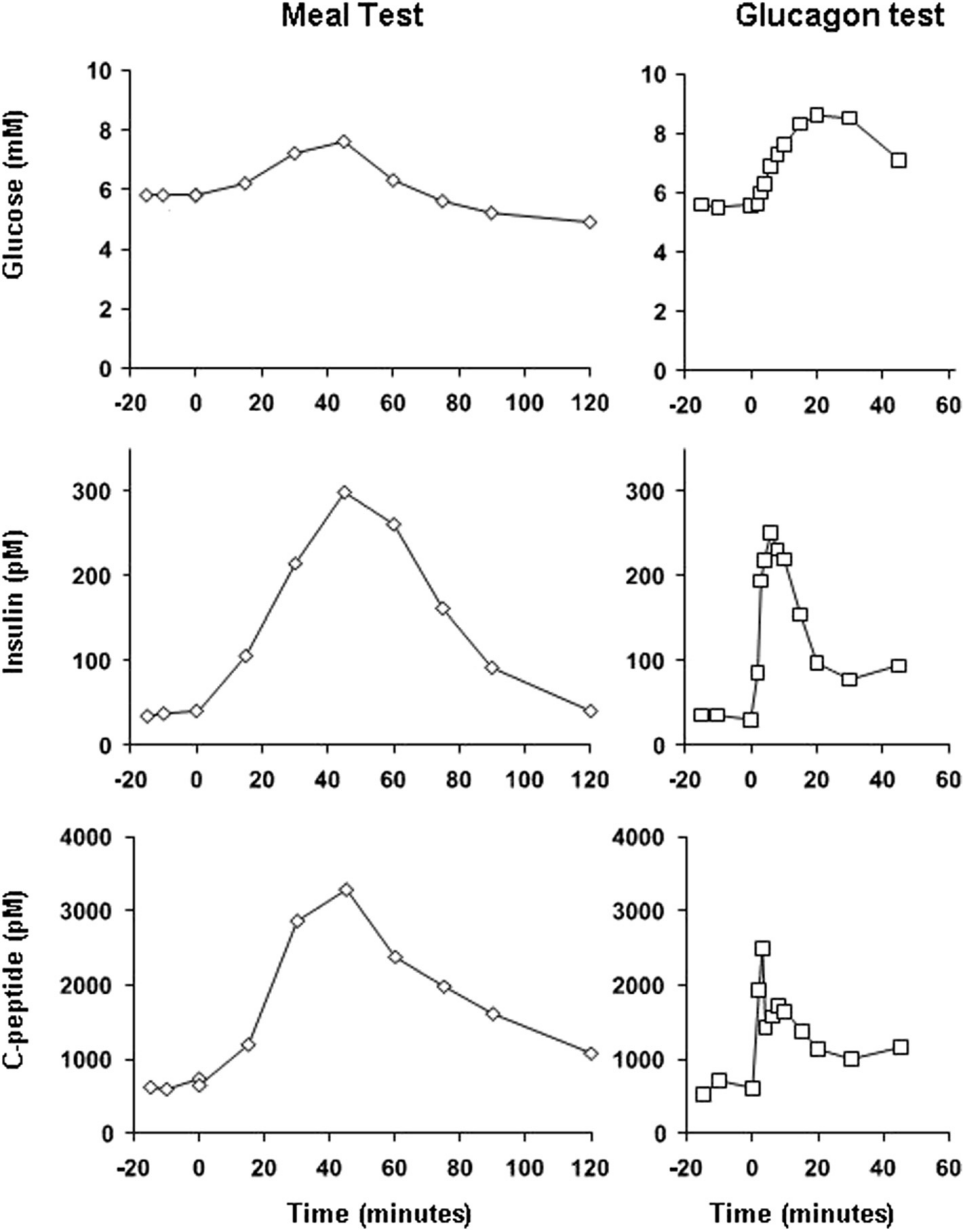
**Meal test and glucagon test.** Plasma glucose (upper panels), insulin (mid panels) and C-peptide (lower panels) concentrations during meal test and glucagon test in a healthy subject with normal glucose tolerance.

No difference in fasting plasma glucose between the 4 experimental days was observed (average fasting plasma glucose: 5.7 mM). Time courses for plasma glucose during day 1 and 2 are shown in Figure 1. During the meal test plasma glucose increased to a peak value of 7.6 mM 45 min following meal ingestion. On day 2 a peak value of 8.6 mM was observed 20 min after injection of glucagon. During the two 2 h hyperglycaemic clamps plasma glucose was elevated to 16.4 mM (GLP-1 clamp) and 14.8 mM (GIP clamp) using 60 ml 50% (w/v) glucose and maintained at mean concentrations of 14.8 mM (range: 12.2-17.6 mM) and 14.6 mM (range: 13.3-17.2 mM), respectively, using a total of 287 g and 287 g of glucose. Following termination of the GLP-1 clamp, plasma glucose concentrations dropped to below 3 mM within minutes and remained here for approximately 1 h in spite of juice and food ingestion. In this period the subject experienced typical symptoms of hypoglycaemia.

### GLP-1 and GIP

During the meal test basal levels of plasma GLP-1 (9 pM) rose to a peak concentration of 19 pM 90 min following initiation of ingestion and returned to basal levels at the end of the meal test (180 min). Basal levels of plasma GIP (6 pM) exhibited a more brisk rise in response to meal ingestion and peak concentration of 145 pM was attained after 30 min (data not shown).

### Insulin and C-peptide

Time courses of plasma insulin and plasma C-peptide responses during day 1 and 2 are presented in Figure 1. No difference in fasting plasma concentrations between day 1 and 2 was observed. As illustrated in Figure 1 the subject responded with normal insulin and C-peptide responses during day 1 (meal test) and day 2 (glucagon test); during day 1, peak concentrations (insulin: 300 pM; C-peptide: 3,278 pM) were attained 45 min following initiation of meal ingestion. During day 2, peak concentrations of 250 pM and 2,483 pM for insulin and C-peptide, respectively, were observed 6 and 3 min following *iv* injection of glucagon.

**Figure 2:**
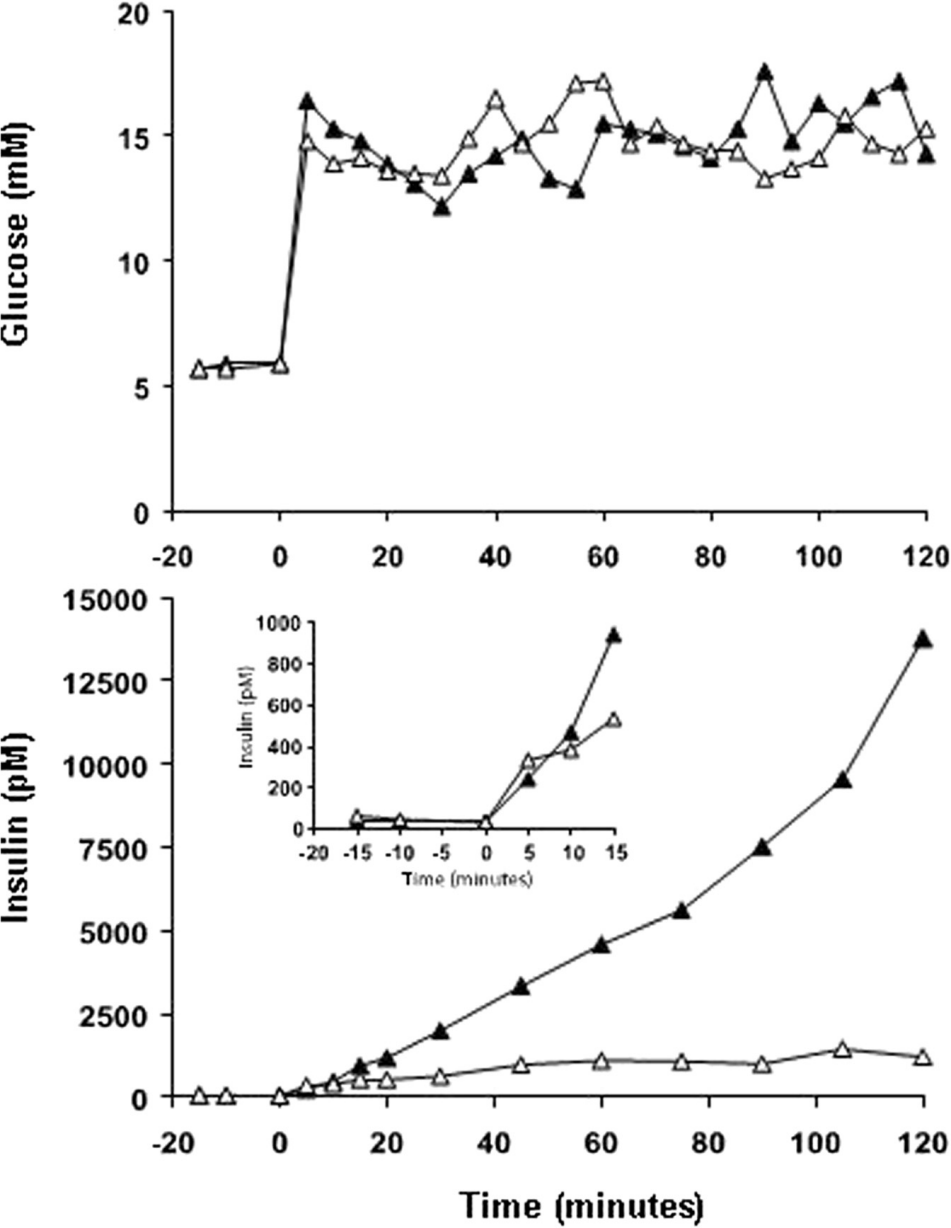
**Hyperglycaemic clamps with GLP-1/GIP.** Plasma glucose (upper panel) and insulin (lower panel) concentrations in a healthy subject with normal glucose tolerance during hyperglycaemic clamps with continuous infusion of glucagon-like peptide-1 (filled triangles) and glucose-dependent insulinotropic polypeptide (open triangles), respectively. The insert in the lower panel illustrates plasma insulin concentrations during the initial 15 min of the two clamps.

During the 15 mM-hyperglycaemic clamp with continuous *iv* infusion of GIP, plasma insulin concentrations increased from a baseline level of 48 pM to a plateau level of 1,163 pM (range: 1,080-1,432 pM (60–120 min)) (AUC_0–120 min_: 104; AUC_0–10 min_: 2.8; and AUC1_0–120 min_: 101 nM × min) (Figure 2).

During the hyperglycaemic clamp with continuous *iv* infusion of GLP-1, plasma insulin concentrations increased almost exponentially from a baseline level of 35 pM to 13,770 pM at the termination of the 2 h clamp (AUC_0–120 min_: 580; AUC_0–10 min_: 2.5; and AUC_10–120 min_: 577 nM × min) (Figure 2). Plasma C-peptide concentrations increased from a baseline of 700 pM to 22,380 pM. Basal plasma proinsulin (8 pM) increased to 602 (at time point 105 min) and 721 pM (at time point 120 min) during this clamp.

## Discussion

With the present case we report, to our knowledge, unprecedented high plasma insulin (13,770 pM) and C-peptide concentrations (22,380 pM) in a healthy subject.

Maximum beta cell secretory capacity has been investigated intensively with the aim of describing beta cell dysfunction in type 2 diabetes. Several tests have been designed to evaluate this parameter including meal tests, the glucagon test [11], the hyperglycaemic clamp test (with or without arginine) and tests combining glucose and non-glucose stimuli such as arginine and GLP-1 [12]. The highest insulin and C-peptide concentrations in healthy subjects reported hitherto were elicited by a test designed to evaluate distinct patterns of insulin secretion including maximal beta cell secretory capacity [12]. This design by Fritsche *et al.* involved a 200 min 10 mM-hyperglycaemic clamp with continuous *iv* infusion of GLP-1 (1.5 pmol/kg/min) starting at 120 min and an arginine bolus of 5 g at 180 min performed in 7 healthy volunteers. Following the arginine bolus mean peak concentrations of insulin and C-peptide amounted to 8,495 pM (±1499 pM, standard error of the mean) and 14,729 ± 1863 pM, respectively. These responses exceeded those seen following arginine given as a bolus during hyperglycaemic conditions (>25 mM glucose) - a test often considered to provoke the maximal insulin response [13] - by more than 100%. Interestingly, the immense beta cell secretory responses in this report and the responses reported by Fritsche *et al.* occurred with a continuous GLP-1 stimulus during hyperglycaemic conditions. Furthermore, there were no signs indicating that the beta cell response in our subject or the responses in the study by Fritsche *et al.* were about to level off towards the end of the hyperglycaemic clamps. Actually, at that point, the insulin curves increased steadily (almost exponentially in the present case). There is no doubt that among the tests used in the present case the 15 mM-hyperglycaemic clamp with concomitant infusion of GLP-1 is the strongest stimulus of beta cell secretion.

What is GLP-1 and how come it is so potent a stimulator of glucose-induced insulin secretion? GLP-1 is a product of the proglucagon gene [14] that, when expressed in mucosal enteroendocrine L cells is processed by prohormone convertase 1/3 to GLP-1, glucagon-like peptide-2 (a key regulator of small intestinal growth) and glicentin. The other incretin hormone GIP is a 42 amino acid-peptide processed (also by prohormone convertase 1/3) from a precursor of 153 amino acids [15] in mucosal enteroendocrine K cells. GLP-1 and GIP are secreted in response to ingestion of nutrients, with lipids and simple carbohydrates being potent stimulators of secretion [1]. In the present case the endogenous incretin hormone responses were evaluated during a meal test (day 1) and the subject exhibited postprandial responses within the normal range.

Specific receptors of GLP-1 and GIP, both belonging to the glucagon subfamily of type 2 G protein-coupled receptors, are found in the pancreatic beta cell plasma membrane [2]. Following binding and subsequent activation of adenylate cyclase, intracellular accumulation of cAMP, closure of ATP sensitive K^+^-channels (K-ATP channels) and elevation of cytosolic Ca^++^ concentrations, mobilisation and exocytosis of insulin containing granules occur [16]. These molecular signalling mechanisms are very similar for the two receptors.

In the present design the doses of incretin hormones used during the hyperglycaemic clamp experiments (1 pmol GLP-1/kg/min and 4 pmol GIP/kg/min) were chosen to obtain maximum beta cell stimulation without causing side effects such as nausea. As illustrated in the insert of Figure 2 these two supraphysiological stimuli resulted in similar first-phase insulin responses (AUC_0–10 min_) whereas the second-phase insulin response (AUC_10–120 min_) with concomitant GLP-1 infusion was almost 6 times greater as compared to the response during concomitant GIP infusion, which did not induce the same exponential rise in insulin secretion as GLP-1. Thus, it seems likely that the mechanistic differences in the insulinotropic power of the two incretin hormones take place during the second-phase insulin response. The mechanisms underlying the second-phase insulin response have been suggested to be due to a signal generated by the K-ATP channel-independent pathway (amplifying pathway) and requires that the beta cell replaces released docked granulas from a reserve pool followed by preparation for exocytosis [17]. The present results suggest that GLP-1 and GIP differ in relation to the effect on the insulin secretory machinery in the beta cell. It is well-established that both GIP and GLP-1 stimulate insulin biosynthesis [4], and thereby provides supplies of insulin for secretion. Additionally GLP-1 may also stimulate second-phase insulin secretion by other means. The abovementioned accumulation of cAMP and closure of K-ATP channels upon stimulation of the GLP-1 receptor and the GIP receptor, respectively, is related to the activation of protein kinase A, which decreases the stimulatory action of ADP at the K-ATP channels. Also, cAMP may act via Epac2 to increase the inhibitory action of ATP at the channel [18], and furthermore, some evidence suggests that cAMP affects K-ATP channel function by up-regulating glucose-dependent mitochondrial ATP production and thereby closing the K-ATP channels [19]. If the GLP-1 receptor is coupled to all of these three pathways, and the GIP receptor is coupled to only one or two, it would explain the superior insulinotropic potency of GLP-1 despite the fact that both incretin hormones are cAMP-elevating agents. Obviously these are speculations that need to be tested mechanistically.

## Conclusion

Here we report unprecedented high plasma insulin and C-peptide responses (elicited by *iv* infusion of GLP-1 during hyperglycaemia) in a healthy subject, and propose potential mechanistic differences underlying the differential insulinotropic effects of the two incretins.

## Consent

Written informed consent was obtained from the patient for publication of this Case Report and any accompanying images. A copy of the written consent is available for review by the Editor-in-Chief of this journal.

## Competing interests

The authors declare that they have no competing interests.

## Authors’ contributions

FKK and AL wrote the manuscript. TV carried out the experiments and reviewed the manuscript. SM, JJH and TK supervised the experiments and reviewed the manuscript. All authors have read and approved the final version of the manuscript.
